# Emerging partner violence among young adolescents in a low-income country: Perpetration, victimization and adversity

**DOI:** 10.1371/journal.pone.0230085

**Published:** 2020-03-06

**Authors:** Rachel Kidman, Hans-Peter Kohler

**Affiliations:** 1 Department of Family, Population and Preventive Medicine, Core Faculty, Program in Public Health, Stony Brook University (State University of New York), Stony Brook, NY, United States of America; 2 Department of Sociology and Population Studies Center, University of Pennsylvania, Philadelphia, PA, United States of America; University of Sao Paulo Medical School, BRAZIL

## Abstract

**Background:**

Intimate partner violence (IPV) is prevalent in high- as well as low-income contexts. It results in a substantial public health burden and significant negative socioeconomic and health outcomes throughout the life-course. However, limited knowledge exists about IPV during early adolescence. This period is critical during the transition to adulthood for at least two reasons: it is when the majority of adolescents in low-income countries first encounter dating, sexuality and partnerships, often with older adolescents or adults, and it is also the period when lifelong patterns of violence and norms about acceptable IPV are formed. The current study is one of the first to measure IPV prevalence among young adolescents in a low-income setting, examine the potential etiology, and investigate relationships with gender ideology, poverty, mental health and childhood adversity.

**Methods:**

We surveyed 2,089 adolescents aged 10–16 in Malawi using standardized instruments. We estimated the prevalence of IPV, and use multivariate logistic regression to test potential correlates.

**Results:**

More than a quarter (27%) of ever-partnered adolescents in Malawi report being victimized. A substantial proportion of both male and female adolescents (15%) report committing violence against their partner. Girls were more likely than boys to report being a victim of sexual IPV (24% versus 8%), and boys more likely to perpetrate such (9% versus 1%). Almost 10% of the sample had both committed and been a victim of IPV. Cumulative childhood adversity (e.g., physical abuse, witnessing domestic violence) was a consistent and strong correlate of IPV victimization (adjusted odds ratio (aOR) 1.30) and of perpetration (aOR 1.35). Depression and PTSD were likewise associated with IPV victimization in the overall sample. Notably, gender ideology was not predictive of either victimization or perpetration, even among boys.

**Conclusions:**

IPV is common for both male and female young Malawian adolescents, and includes both victimization and perpetration. IPV compounds other adversities experienced by adolescents in this low-income setting, and it is rarely alleviated through help from the health system or other formal support. These findings underscore the need to intervene early when interventions can still break destructive pathways and help foster healthier relationships. This focus on early adolescence is particularly critical in low-income countries given the early onset and rapid pace of the transition to adulthood, with sexual activity, dating and partnership thus being common already in young adolescence. Promising interventions would be those that reduce violence against or around children, as well as those that reduce the impacts of such trauma on mental health during adolescence.

## Introduction

Globally, over 30% of women have experienced intimate partner violence (IPV) [1]. IPV results in a substantial public health burden, including homicide, injury, depression, and HIV infection [2–5]. There is global movement to combat IPV. This commitment is most recently reflected in the Sustainable Development Goals to reduce IPV (SDG 5.2.1) and to end violence against children (SDG 16.2). However, a better understanding of the emergence and etiology of IPV is needed to guide effective strategies for meeting these goals.

The highest incidence of IPV is generally reported during late adolescence and early adulthood [6]. In a study across 81 countries, 29% of ever-partnered adolescent girls (aged 15–19) reported they had already experienced IPV [7]. Studies typically only include women 15 and older [e.g., 7, 8]. Thus, we know very little about the emergence of IPV during early adolescence (i.e., 10–14 years) when many adolescents–and especially adolescents in low-income countries where the transition to adulthood begins early and progresses rapidly [9]–experience their first romantic or sexual relationship within the context of dating [10]. Importantly, experiencing IPV may have different developmental significance during early adolescence. Early to mid-adolescence is a period of rapid brain development, distinct from later adolescence (6), and a particularly sensitive period for social and emotional learning (4). A positive sense of self; emotional and physical safety; and engagement in learning are key components to healthy development during early adolescence [11]. As adolescents internalize experiences of IPV, however, they are forming harmful gender roles and attitudes that become part of their identity [12]. In this way, early romantic and sexual relationships can influence the quality of later relationships and are important determinants of lifelong health [9, 11].

Research and intervention typically overlook this period (4, 5). Estimates from the Global School-based Student Health Surveys (GSHS) are an exception that shed light on IPV in early adolescence in low- and middle-income countries. In four select countries, the GSHS asked adolescents as young as 13–15 years old about their experiences of violence; past year physical IPV ranged from 6%-19% for girls and 8–23% for boys [13]. The Violence Against Children surveys, conducted in a growing number of countries, also collects data on IPV among adolescents aged 13–17 years old. In Malawi, past year physical IPV estimates are lower: only 4% for girls and 1% for boys. Just under a quarter of the girls surveyed reported past year sexual abuse, with 26% of those naming a romantic partner as the perpetrator [14].

Adolescence is likewise a time when both boys and girls first become perpetrators of violence. For example, in South Africa, 40% of adolescent boys aged 15–19 years old report perpetrating sexual or physical IPV in the past year [15]. There is far less data on female perpetration, but what is available suggests that girls often perpetrate violence at rates similar to–or even greater than–boys [16–20]. A 32-country study, for example, found female university students were more likely to be physically violent towards their partner compared to male students [18]. This was not true, however, in a recent study of four African countries: 6–7% of adolescent girls and young women (aged 13–24) reported perpetrating physical violence against partner; for boys and young men the range was 8–19% [21], suggesting context matters. In Malawi 17% of girls 17% of girls 38% of boys age 13–17 years old report ever perpetrating physical or sexual violence against a partner [14].

A substantial body of work has examined predictors for IPV, with the hope of identifying intervention targets. Studying the period of early adolescence is particularly useful in this regard, as many potential causes of IPV are rooted in these early years. A common approach is to focus on the role of family conflict [22]. We have learned, for example, that individuals who report child abuse are much more likely to perpetrate IPV [23, 24]. Likewise, a 2018 review found that children who witness parental IPV were up to four-fold more likely to perpetrate IPV as adults [25]. In retrospective studies, cumulative childhood adversity predicts physical IPV among youth [26]. There is evidence that psychological distress, including depression and posttraumatic stress disorder (PTSD), may mediate the relationship between family conflict and victimization [27]. However, the influence of family conflict (and causal mechanisms) may differ substantially by country context, and previous authors have highlighted the lack of research from low- and middle-income countries (LMIC) [25]. For instance, there is important variation in corporal punishment from country to country [28], which may influence the etiology of IPV [29]. The evidence base continues to primarily reflect high-income contexts [e.g., 30], though investigations in LMIC are slowly growing. In the African study referenced above, being a victim of childhood violence increased the odds of ever perpetrating violence in all four countries, with the odds ratio ranging from five to seven [21]. In this study, however, violence perpetration outcome included but was much broader than violence towards an intimate partner.

Other studies of etiology adopt the framework of feminist theory. Such investigations–and resulting interventions—focus on how gender inequities shape violence [31, 32]. During adolescence, IPV can be a product of and reinforce gender norms. We know adolescence is a critical period for gender socialization [12]. We also know that adolescents have a strong desire to conform to perceived norms, and thus are particularly sensitive to inequitable gender expectations [33]. Several, but not all, studies have shown that more inequitable gender role attitudes increase the risk of IPV perpetration by adolescent boys [34]. There is great variability in the cultural views of women, which may explain some of the observed differences in adolescent IPV between countries [35]. It has been suggested that a feminist lens is even more relevant in LMIC, where women often have less autonomy, less political and economic power, fewer legal protections, and more restrictive gender expectations [36, 37]. Thus in such settings, the relative contribution of gender norms to the development of IPV may be greater [38].

Of course, these perspectives aren’t mutually exclusive. IPV most likely results from multiple causal factors [18, 39]. The strength of risk factors for IPV are likely to differ by country of residence (e.g., internalizing problems) [40], as well as by stage of the transition to adulthood. Extreme poverty, for example, is one of the most important determinants of IPV victimization among young girls in some settings [e.g., 41], though not in others [42]. These theoretical perspectives, however, often lead to very different intervention approaches. Understanding which factors are most prominently associated with IPV in various settings can help prioritize prevention targets. This paper therefore analyzes data from a large cohort of young adolescents (aged 10–16 years) in Malawi, a low-income country, in order to answer the following questions: 1) what is the prevalence of IPV victimization and perpetration among ever-partnered adolescents? and 2) which childhood and adolescent factors are correlated with IPV victimization and perpetration?

## Materials and methods

### Sample and setting

This study took place in rural Malawi. Approximately 80% of Malawians live below the international poverty line (US$1.90 per day), and the rates are even higher in rural areas [43]. HIV/AIDS remains a persistent challenge: prevalence among Malawian adults is 7.5% [44]. The sample frame was derived from the Malawi Longitudinal Study of Families and Health (MLSFH) [45]. The MLSFH was established in 1998 to better understand the lives of rural individuals in a low income context, and to specifically focus on health, HIV, and demographic change. The original MLSFH cohort was selected to broadly represent the rural population (≈84% of Malawi’s population) and is located in three districts (Balaka, Mchinji, and Rumphi). The MLSFH has subsequently undertaken multiple rounds of data collection, yielding 20 years of rich data on individual adults and their households. For this study, we created a new, early adolescent cohort by selecting adolescent children of MLSFH respondents. Specifically, for each adult MLSFH respondent who completed a household roster in either 2008 or 2010, we selected children projected to be age 11–15 in 2017. To create sibling matches in households with only one child aged 11–15 at baseline, we extended the age range by one year in both directions and enrolled the child closest in age to the index child. This produced a sample frame of adolescents aged 10–16, a critical age-range in which to assess emerging IPV. We obtained Institutional Review Board approval from Stony Brook University and the National Health Science Research Committee in Malawi.

### Data collection

Data collection occurred between August 2017 and June 2018. A total of 2,089 adolescents were located and interviewed in their local language (Chichewa, Chiyao or Chitumbuka) by a trained interviewer from the same district. 1,787 were located at or near the homes of the original adult MLSFH respondents; an additional 114 were traced to new homes within the same cluster of villages. Of those who had migrated further, we attempted to locate those still residing within their home district or who had moved to a major city. We captured an additional 262 respondents through such migrant tracing. Adolescents provided written assent after guardian consent. There were only 13 refusals. All face-to-face interviews were conducted privately at the adolescent’s home; interviews were halted immediately if privacy could not be ensured. In addition to IPV, surveys asked about childhood adversity; social, emotional and cognitive impairment; and indicators of early sexual risk taking. The adolescent’s caregiver completed a separate survey on household characteristics and wellbeing. Interviewers were trained to monitor distress during the interview; the field team operated a 24-hour hotline during and for two months after the study period, and protocols were in place to arrange any necessary support.

### Measures

*Intimate partner violence* was measured among those respondents who either reported 1) sexual debut (i.e., prior sexual intercourse with anyone) and/or 2) a current or past romantic partner. Questions were adapted from the WHO’s Violence Against Women instrument (VAWI) [6]. While originally designed to capture victimization among females, the VAWI has been adapted by others to capture perpetration in Tanzania [46] and in South Africa [47]. Moreover, it has shown acceptable internal consistency for both victimization and perpetration among young men and women [46]. A subset of the VAWI questions have also been used by the VACS initiative to measure perpetration in multiple countries, including Malawi [48]. All questions ask about violence pertaining to a “current boyfriend/girlfriend or any other partner.” Six questions assessed lifetime physical IPV (e.g., having been slapped, kicked); two assessed lifetime sexual IPV (e.g., forced or coerced intercourse); and three assessed emotional violence (e.g., threatened with harm, humiliated). Respondents were considered to have experienced IPV if they answered any question affirmatively, with separate indicators created for each type of IPV and for total IPV exposure. The VAWI questions were adapted to capture lifetime IPV perpetration, with the same coding scheme applied. We also created a variable labeled “dual IPV” which reflects whether an individual reported both IPV victimization and perpetration, though we have no data on whether this occurred with the same partner. Finally, help-seeking was assessed with the question “When you've been hurt—physically or sexually—have you ever sought help?” For affirmative responses, they were asked who they sought help from.

We focus our investigation of correlates on common risk factors for IPV in older adolescents and adults, though with the additional caveat that much of this evidence comes from high-income contexts. Potential risk factors include adverse childhood experiences, trauma symptoms, depression, and gender attitudes [30, 39, 49–51]. In LMIC, economic hardship may also play an important role [52]. We used the Adverse Childhood Experiences International Questionnaire (ACE-IQ) [53] to capture lifetime adversity. The ACE-IQ includes 13 domains of individual (e.g., physical abuse), family (e.g., witnessing domestic violence), peer (bullying) and community (e.g., gang violence) influences. We use the frequency coding scheme to create a cumulative measure (0–13). We measured emotional states by using the Posttraumatic Stress Disorder Scale (PTSD-8; Cronbach’s alpha = 0.77) [54] and the Beck Depression Inventory (Cronbach’s alpha = 0.90; depression was analyzed using the pre-established cut-point for moderate/severe depression) [55]. We used five questions on attitudes towards wife beating (from the Demographic Health Surveys; e.g., “In your opinion, is a husband justified in hitting or beating his wife if she goes out without telling him?”) and five questions on female autonomy (from early rounds of the MLSFH; e.g., “Is it acceptable for a woman to go to the local health center without informing her husband?) to capture gender ideology; these formed a scale ranging from 0–10 (Cronbach’s alpha = 0.56) [56]. Caregivers were asked to complete a checklist of potential household assets. We then created a socioeconomic status (SES) index by summing assets, each weighted by the inverse of the proportion of the population owning that particular asset. For these analyses, we divided the index into SES quintiles. Additional covariates included age (measured continuously), gender, and region.

### Analyses

We estimated the prevalence of IPV victimization and perpetration among ever-partnered adolescents, both for the total sample and by gender. Subgroup differences were tested in logit models with either gender or age group as the only predictor, as appropriate. Help-seeking was rare, and thus frequency data are presented aggregated across genders. Finally, we used multivariate logistic regression to test associations between potential risk factors and IPV. Two sets of models were created: the first examine the association between individual risk factors and IPV, controlling only for age, gender, and region, and using the cluster command to adjust standard errors for correlation at the caregiver level. A second set examined all potential risk factors simultaneously. The same approach was then applied to the data stratified by gender, as past research has shown differences in risk factors by gender [39]. Analyses were run using Stata v13.

### Results

In our sample of rural adolescents aged 10–16 years old, over a quarter (28%, n = 587) reported a romantic or sexual partnership. Not surprisingly, partnerships were far more common among older adolescents (52% of 15–16 year olds compared to 19% of 10–14 year olds). Thus the mean age of the ever-partnered cohort was 14 (SD 1.5). Partnerships were also more common among boys (32%) than among girls (24%). Marriage and cohabitation, however, were only reported among girls (n = 19). Unless otherwise noted, all our subsequent results on IPV pertain to ever-partnered adolescents.

### Prevalence of IPV victimization and perpetration

Over a quarter of ever-partnered adolescents, and 8% of all adolescent respondents, reported being a victim of physical, sexual or emotional IPV in their lifetime (Table 1), with only modest differences by gender. There was more variation in the type of violence experienced, most notably with 24% of ever-partnered girls and only 8% of boys reporting sexual victimization. A lower but still substantial proportion (15%) of adolescents reported perpetrating violence, with statistically significant differences between girls (10%) and boys (18%). Again, key differences emerge by type: boys were more likely to perpetrate physical (8% versus 3% of girls) and sexual IPV (9% versus 1% of girls); whereas the two groups were equally likely to perpetrate emotional IPV (7%). Overall, 9% of the sample indicated they had both committed and been a victim of IPV. Table 1 further breaks down reports of IPV by age group. For those who had ever been in a relationship, there were no statistical differences in the reported physical, sexual or emotional IPV between participants in very early adolescence (age 10–14) and participants in middle adolescence (15–16) (data not shown).

**Table 1:** Prevalence of lifetime IPV victimization and perpetration among ever-partnered adolescents, by age.

Age 10–16 (n = 586)				
	**All**	**Boys**	**Girls**	**p-value**
**Victimization**				
Physical IPV	11%	13%	8%	0.070
Sexual IPV	15%	8%	24%	0.000
Emotional IPV	11%	12%	9%	0.321
Any IPV	27%	24%	31%	0.057
**Perpetration**				
Physical IPV	6%	8%	3%	0.024
Sexual IPV	6%	9%	1%	0.001
Emotional IPV	7%	7%	7%	1.000
Any IPV	15%	18%	10%	0.007
**Dual IPV**	9%	10%	7%	0.175
**Age 10–14 (n = 296)**				
	**All**	**Boys**	**Girls**	**p-value**
**Victimization**				
Physical IPV	11%	11%	9%	0.578
Sexual IPV	14%	8%	25%	0.000
Emotional IPV	11%	11%	12%	0.856
Any IPV	28%	23%	36%	0.020
**Perpetration**				
Physical IPV	7%	8%	5%	0.250
Sexual IPV	5%	7%	1%	0.045
Emotional IPV	7%	5%	7%	0.761
Any IPV	15%	17%	11%	0.177
**Dual IPV**	9%	9%	9%	0.997
**Age 15–16 (n = 290)**				
	**All**	**Boys**	**Girls**	**p-value**
**Victimization**				
Physical IPV	11%	14%	7%	0.047
Sexual IPV	15%	8%	23%	0.000
Emotional IPV	10%	12%	7%	0.126
Any IPV	27%	26%	28%	0.676
**Perpetration**				
Physical IPV	5%	8%	2%	0.049
Sexual IPV	7%	12%	1%	0.003
Emotional IPV	7%	8%	7%	0.749
Any IPV	16%	21%	10%	0.012
**Dual IPV**	9%	12%	5%	0.063

### Help-seeking among victims

Of the adolescents who reported being a victim of IPV, a quarter (n = 40) reported seeking help. Help-seeking was slightly more prevalent among female compared to male victims (29% versus 22% respectively). It also varied slightly by type of victimization: 21% of those reporting physical IPV, 29% of those reporting sexual IPV, and 31% of those reporting emotional IPV also reported seeking help. Adolescents most commonly reported turning to friends (n = 21) and family (n = 10), with very few seeking care from a health facility (n = 3) or other formal support.

### Childhood and adolescent correlates of IPV

The distribution of potential correlates is displayed in Table 2; their association with IPV is displayed in Table 3. The most consistent correlate of IPV victimization was childhood adversity. For girls, adversity exhibited an association with both victimization and perpetration (aOR 1.45 and aOR 1.36 per increase in a single ACE respectively). For boys, adversity was a slightly more powerful predictor of perpetration (aOR 1.23 for victimization and aOR 1.36 for perpetration). Both PTSD and depression also exhibited a strong correlation with IPV victimization (aOR 1.70 and 1.79 respectively), but lost statistical significance in some gender-stratified analyses. For girls only, depression remained a significant correlate of IPV victimization (OR 2.42) and perpetration (OR 2.90). Neither gender ideology nor poverty was associated with IPV.

**Table 2:** Distribution of potential psychosocial correlates among ever-partnered adolescents age 10–16.

Characteristic	**All**	**Boys**	**Girls**
	Mean (SD)	Mean (SD)	Mean (SD)
ACE score	3.11 (1.94)	2.80 (1.92)	3.30 (1.96)
Gender ideology	5.11 (1.61)	5.09 (1.53)	5.13 (1.71)
	N (%)	N (%)	N (%)
PTSD	88 (15%)	43 (13%)	45 (19%)
Depression	119 (20%)	64 (19%)	55 (23%)
SES quintile			
Lowest	181 (32%)	108 (32%)	73 (31%)
Second	88 (15%)	45 (12%)	43 (18%)
Third	116 (20%)	66 (20%)	50 (21%)
Forth	103 (18%)	67 (20%)	36 (15%)
Highest	89 (15%)	52 (15%)	37 (15%)

**Table 3:** Correlates of lifetime IPV victimization and perpetration among ever-partnered, young adolescents (age 10–16)†.

	**IPV Victimization**		**IPV Perpetration**	
	Bivariate OR (95% CI)	Adjusted OR (95% CI)	Bivariate OR (95% CI)	Adjusted OR (95% CI)
**All**				
ACE score	1.32*** (1.20, 1.46)	1.30*** (1.17, 1.43)	1.36*** (1.21, 1.53)	1.35*** (1.19, 1.52)
PTSD	2.04** (1.24, 3.32)	1.70* (1.00, 2.90)	1.63 (0.87, 3.08)	1.36 (0.70, 2.64)
Depression	2.10*** (1.37, 3.21)	1.79* (1.12, 2.85)	2.01** (1.20, 3.37)	1.55 (0.86, 2.77)
Gender ideology	0.99 (0.88, 1.11)	1.01 (0.89, 1.14)	0.92 (0.80, 1.06)	0.94 (0.80, 1.10)
SES quintile	0.96 (0.83, 1.10)	0.97 (0.84, 1.12)	0.97 (0.81, 1.17)	0.98 (0.81, 1.18)
Age (in years)		0.99 (0.86, 1.13)		1.03 (0.88, 1.21)
Female gender		1.30 (0.97, 1.93)		0.37*** (0.22, 0.67)
**Boys**				
ACE score	1.24*** (1.09, 1.41)	1.23** (1.08, 1.41)	1.36*** (1.16, 1.59)	1.36*** (1.16, 1.60)
PTSD	1.68 (0.83, 3.40)	1.50 (0.73, 3.12)	1.26 (0.54, 2.97)	1.15 (0.47, 2.85)
Depression	1.69 (0.93, 3.06)	1.44 (0.76, 2.71)	1.38 (0.73, 2.61)	1.08 (0.52, 2.25)
Gender ideology	0.94 0.79, 1.12)	0.99 (0.82, 1.19)	0.92 (0.77, 1.09)	0.91 (0.75, 1.12)
SES quintile	0.97 (0.81, 1.16)	0.96 (0.79, 2.52)	0.92 (0.75, 1.13)	0.90 (0.73, 1.12)
Age (in years)		1.02 (1.86, 1.21)		1.03 (0.86, 1.23)
**Girls**				
ACE score	1.45*** (1.24, 1.69)	1.41*** (1.20, 1.65)	1.36** (1.13, 1.62)	1.32** (1.10, 1.58)
PTSD	2.30* (1.14, 4.62)	1.73 (0.75, 3.99)	2.49 (0.95, 6.51)	1.92 (0.69, 5.39)
Depression	2.72** (1.45, 5.08)	2.42** (1.18, 4.98)	4.09*** (1.73, 9.66)	2.90* (1.08, 7.81)
Gender ideology	1.03 (0.88, 1.20)	1.03 (0.86, 1.23)	0.91 (0.72, 1.15)	0.96 (0.72, 1.28)
SES quintile	0.95 (0.76, 1.19)	0.99 (0.77, 1.27)	1.10 (0.75, 1.61)	1.14 (0.77, 1.67)
Age (in years)		0.95 (0.75, 1.19)		1.07 (0.75, 1.52)

†bivariate models examine a single potential covariate controlling for age, gender, and region; adjusted models include all potential correlates simultaneously.

*≤p = .05

**≤p = .01

***≤p = .001

### Discussion

In rural Malawi, a substantial proportion (27%) of young, ever-partnered adolescents aged 10–16 report having been victimized in their sexual and romantic relationships, though few sought out any type of help. A lesser but still notable proportion (15%) report committing violence against their partner. Our findings have important implications for the timing, targeting and approach to IPV prevention.

### First, setting the stage for lifelong healthy relationships begins in early adolescence

To date, most IPV prevention initiatives have been developed and tested among older populations in high-income contexts [49]. Previous studies have documented prevalent IPV in cohorts aged 15–19 years, and used this data to suggest that prevention efforts begin before age 15 [15, 35, 57]. We are one of the first studies to examine IPV during early adolescence, and find IPV is equally common in relationships among younger adolescents (aged 10–14). This underscores the need to intervene earlier in the life course, when we can still break destructive pathways and foster healthier relationship [17].

### Second, responses need to acknowledge that girls are perpetrators of IPV–particularly of emotional abuse

Female perpetration (10% among ever-partnered girls in this study) is not a unique finding [58–60], though we extend it to a younger age group and add to the limited literature from a low-income context. Moreover, we are one of the few studies to examine emotional violence; we find that this is the form of IPV perpetration most often employed by girls. The accumulating evidence on female perpetration contradicts the often implicit bias in programs that treat women and girls primarily as victims [for examples of interventions see 61, 62]. Prevention approaches that recognize and respond to female perpetration and its unique drivers might be more effective. We found many girls both commit and are a victim of violence. From studies in high income contexts, we know that when violence is reciprocal, women are more likely to be severely injured [63]. Thus recognizing and addressing female perpetration–perhaps most specifically related to emotional violence—is critical to the safety of both partners.

School-based interventions are one of the more promising strategies for reducing adolescent IPV [49]. These interventions reach both boys and girls during adolescence, and typically foster communication and negotiation skills appropriate for both genders. However, intervention effects can differ by gender: some are equally effective in reducing perpetration by boys and girls; others are less effective [64] or not effective among girls [65]. More research is needed to understand the reasons for this heterogeneity, to understand which types of interventions are best at reducing emotional violence, and to implement gender-neutral or gender-specific programming accordingly [66].

### Third, there is a need to diversify the theoretical approaches to IPV prevention

The findings from this study provide important insights into the mechanisms driving IPV, and thus what strategies may be effective in reducing IPV. This study did not find evidence that adolescents who espoused more inequitable gender ideologies were more likely to perpetrate violence. This adds to the already mixed literature on the subject, with several reports highlighting heterogeneity in the relationship across countries [15, 67, 68]. Fleming et al., for example, found inequitable gender attitudes predicted male perpetration in only two (Bosnia and Mexico) of eight countries studied [68]. Similarly, Peitzmeier et al. found gender attitudes predicted IPV perpetration among urban, adolescent males in South Africa and China, but not in the United States or India [15]. Moreover, while boys were more commonly perpetrators of violence in our study, this was not exclusively their domain: 10% of girls reported committing violence against a partner. In the previous Violence Against Children study in Malawi, perpetration among 13–17 year old girls was even higher (17%). If girls are often perpetrators–and boys are victims–this challenges us to rethink how we combat IPV. Feminist theory [31] guides many frameworks on IPV, and thus the prevailing intervention focus is on changing unequal power dynamics [36, 66]. Our finding suggests that gender inequality–though prominent in Malawi—may not be the core cause of IPV in this context.

Our findings indicate that childhood adversity may be an important driver of IPV. The associations between indicators of adversity and IPV were remarkably similar for both for victimization and perpetration, and for both boys and girls. This adds to a robust literature in high-income countries linking childhood adversity, including witnessing domestic violence, to later IPV experiences [26, 59, 69]. A similar literature is emerging in LMIC countries, including in Malawi and South Africa [14, 51]. We extend this work to a new developmental period (early adolescence), showing that childhood adversity has a strong influence on the quality of formative relationships. This is, moreover, one of very few studies that examine risk factors for girls’ perpetration of violence, particularly sexual violence [70].

The next step will be to better understand the pathways by which ACEs affect IPV. For instance, chronic stress from ACEs may rewire the brain in ways that make it more difficult to control anger and impulses [71]. If so, trauma-focused care and support during adolescence might help mitigate the impact of adversity on later IPV behaviors [72]. Alternatively, the mechanism may involve social learning [73]: when violence is modelled at home, adolescents may learn that it is socially-acceptable and enact violence in their own relationships. In this case, adolescents may benefit from learning more productive ways to handle conflict [74, 75].

Either way, IPV prevention could start in childhood. Parenting programs, for example, show potential to prevent childhood violence and reduce IPV in high income contexts, and are currently being testing in LMIC [76, 77]. To accelerate these efforts, the WHO published the INSPIRE package outlining seven evidence-based interventions to prevent childhood violence [78]. While these represent an excellent starting point, some strategies (e.g., economic strengthening) have failed to show an impact on family violence in low-income countries [79]. Thus, all strategies need to be carefully adapted and tested in low-income settings. Malawi is already taking the first steps: it has conducted its own study of violence against children and young women, identified prevention approaches across multiple sectors, and has crafted related action plans [80]. Moving forward, it will be critical to work with the government to ensure strategies are properly resourced, adapted and evaluated, in anticipation of eventual scale-up to reach children across Malawi.

### Fourth and finally, our findings are a reminder that boys need equal access to victim support

For many years, research and services implicitly assumed that women were primarily victims and men were almost exclusively perpetrators of IPV [81, 82]. This approach has been challenged more recently [18], yet male victimization is still rarely addressed in LMIC. In our study, more boys reported physical and emotional victimization than did girls. Help-seeking was uniformly low. Indeed, only a handful of either gender had ever sought care from a health facility or other formal support service. In part, victim services have been primarily geared towards women because they are more likely to be severely injured or killed [83, 84]. While injuries may be lower for boys, their greater exposure to emotion violence may have serious psychological consequences. Future work with adolescents should collect data on the health impact by gender in order to appropriately respond to their needs. In the meantime, providers need to see boys as potential victims and survivors of IPV, include them in their outreach and messaging, and work to destigmatize help-seeking among boys.

### Strengths and limitations

This is one of the first studies to characterize IPV victimization and perpetration among a cohort of very young adolescents in a low-income context. We concentrate on an age when lifelong expectations about romantic relationships are forming. In focusing on adolescents, we can also more accurately measure past childhood experiences and concurrent adolescent risk factors, and thus better highlight factors associated with IPV. Moreover, the population is unique in being composed of black Malawians growing up primarily in rural poverty–a sample rarely captured in the interpersonal violence literature. While the original MLSFH cohort was largely representative of rural Malawi when it was created in 1998, we cannot speak definitively to its generalizability two decades later. Other study strengths include the use of standardized measures to capture physical, sexual and emotional IPV; adaption of the same measure to capture perpetration among both girls and boys; and a sample size large enough to investigate correlates of relatively rare events.

The study’s cross-sectional nature, however, precludes causal interpretations. For example, we cannot make any inferences about whether depression is a predictor or consequence of IPV, which has implications for prevention efforts. A follow-up of this cohort is forthcoming in 2020, and the longitudinal data measuring IPV at older ages will be able to better capture risk factors and IPV trajectories. Another limitation is that while the study assessed both victimization and perpetration, it did not collect information on whether these occurred together (escalading reciprocal violence), were unique episodes, or involved acts of self-defense [85]. In this same vein, the study did not collect information on the severity or consequences of violence, which show greater differences by gender [83, 84].

One important challenge to any study of IPV is that we rely on the self-report of sensitive experiences [86]. This may result in social desirability bias, where adolescents under-report behaviors that are viewed in a negative light. In studies of IPV among U.S. university students, however, a measure of social desirability accounted for very little of the variance in IPV reports by women [87]. Moreover, we did not find substantial item non-response, which would be expected if the respondents felt they were being asked about particularly sensitive experiences [88]. We had 586 adolescents complete 21 questions assessing lifetime IPV victimization and perpetration; there were only two instances where a question was skipped.

We note too that this study used face-to-face interviews. While there has been some suggestion that computer-assisted self-interviews (which theoretically provide greater privacy) might yield more accurate data on sensitive behaviors [89], a meta-analysis of 15 studies in LMIC did not find evidence that the newer technology increased reporting of sexual behavior [90]. In Malawi specifically, a previous longitudinal study of adolescents found that that face-to-face interviewing produced more consistent reporting on sexual behaviors compared to computer-assisted self-interviews [91]. Importantly, any social desirability bias is most likely to result in under-reporting [86]. The urge to under-report would be greatest for the most deviant behaviors [92]. Thus, we’d expect perpetration to be under-estimated, even more so than victimization.

Recall bias is another challenge to self-report. For example, a study of adolescents and young adults in Malawi found that the prevalence of self-reported lifetime sexual violence plummeted after age 18, most likely because young adults quickly forgot about much earlier episodes [93]. In this study, adolescents have only recently entered into romantic or sexual partnerships, and thus have a relatively short recall period regarding IPV. This likely adds to the accuracy and value of estimates conducted during adolescence, as compared to retrospective recall of the period by adults.

## Supporting information

S1 Data (DTA)
